# Impact of acupuncture treatment on the lumbar surgery rate for low back pain in Korea: A nationwide matched retrospective cohort study

**DOI:** 10.1371/journal.pone.0199042

**Published:** 2018-06-12

**Authors:** Wonil Koh, Kyungwon Kang, Yoon Jae Lee, Me-riong Kim, Joon-Shik Shin, Jinho Lee, Jun-Hwan Lee, Kyung-Min Shin, In-Hyuk Ha

**Affiliations:** 1 Jaseng Spine and Joint Research Institute, Jaseng Medical Foundation, Seoul, Republic of Korea; 2 Clinical Research Division, Korea Institute of Oriental Medicine, Daejeon, Republic of Korea; 3 University of Science & Technology (UST), Korean Medicine Life Science, Campus of Korea Institute of Oriental Medicine, Daejeon, Republic of Korea; Stanford University School of Medicine, UNITED STATES

## Abstract

**Introduction:**

Low back pain (LBP) is a globally prevalent disorder with high social significance. Invasive surgical procedures are increasingly being used to treat LBP despite a lack of solid evidence supporting their long-term benefits. This nationwide retrospective cohort study investigated the association between acupuncture treatment and lumbar surgery rate in patients with LBP.

**Methods:**

Using the National Health Insurance Service Sample Cohort Database for 2002–2013, we identified newly diagnosed LBP patients in Korea between 2004 and 2010 and divided them into an acupuncture group and control group according to whether or not they received acupuncture. Propensity scores based on age, sex, income, and Charlson Comorbidity Index were matched between the two study groups. The lumbar surgery rate in the two years following the first visit (control group) or the first acupuncture session (acupuncture group) was calculated. In addition to the overall analysis, stratified analyses were also conducted in different age, sex, and income strata. Sensitivity analyses were further performed using varying definitions of acupuncture treatment.

**Results:**

After matching, 130,089 subjects were included in each study group. The lumbar surgery rate was significantly lower in the acupuncture group than in the control group (hazard ratio [HR] 0.633, 95% confidence interval [CI] 0.576–0.696). Decrease in HR was consistently observed in the acupuncture group for all age strata, except for patients in their 20s (HR 1.031, 95% CI 0.804–1.323). HR for lumbar surgery tended to be further reduced in the older age groups upon acupuncture treatment, with no apparent sex-related differences. Lowered HR in the acupuncture group was continuously observed across all income groups; the higher income group showed a tendency of greater decrease. Sensitivity analyses showed that the number of acupuncture sessions had no major impact on the likelihood of lumbar surgery, but also that more intensive acupuncture treatment was associated with further reduction in lumbar surgery rates.

**Conclusion:**

The present results found that administration of acupuncture treatment is associated with lower lumbar surgery rates for LBP patients in Korea. Prospective studies are warranted in the future to further investigate the effect of acupuncture treatment on lumbar surgery incidence.

## Introduction

Low back pain (LBP) is one of the most prevalent disorders worldwide. According to the World Health Organization, six or seven out of every ten individuals will experience at least a single episode of LBP in their lifetime [1]. A review of 165 studies in 54 countries reported that the point prevalence of LBP was 11.9 ± 2.0% and that the one-month period prevalence was 23.2 ± 2.9% [2]. Furthermore, a global analysis using years lived with disability (YLD) showed that LBP was the leading cause of disability, incurring 57.6 million YLDs, and a similar analysis on disability-adjusted life years (DALY) found that LBP, together with neck pain, was one of the three most common causes for DALYs [3, 4].

The treatment options for LBP include physical therapy, exercise therapy, manual therapy, education, cognitive behavioral therapy, pharmacologic therapy, and invasive procedures [5]. Clinical guidelines recommend surgery for LBP only in specific cases, e.g., after failure of more than two years of conservative treatment. Spinal fusion has no increased benefits over intensive rehabilitation for non-radicular LBP; open or microscopic discectomy may confer short-term benefits in terms of pain reduction and improved function, but long-term benefits are elusive [5–8]. Undoubtedly, surgery is not recommended for non-specific LBP [9]. The benefits of spinal surgery have not been firmly established, and such procedures are still associated with considerable financial cost [10] and risks, such as adjacent segment degeneration [11, 12], re-operation [13], and failed back surgery syndrome [14]. Nevertheless, the lumbar surgery rate continues to rise worldwide [15–17].

Treatments that are outside the scope of modern medicine are commonly referred to as complementary and alternative medicine (CAM), the best known of which are acupuncture, manual therapy, yoga, and herbal medicine [18–20]. Acupuncture, in particular, is regarded to modulate pain through regulation of connective tissue [21], nervous system fibers [22], and endogenous neuropeptides [23]. Systematic reviews and trials found that acupuncture alleviates chronic LBP and improves function when used alone or in combination with other therapies [24–26], and that it decreases the societal and medical costs of LBP [27]; likewise, it is being recommended for conservative treatment of LBP in the updated 2017 American College of Physicians’ guideline [28]. The subject yet remains controversial as the National Institute for Health and Care Excellence guideline for LBP recommends against its use [29], whose judgement has also been criticized [30, 31]. Synthesis of ten clinical practice guidelines concluded that acupuncture should be judiciously added onto usual care for additional symptomatic relief of chronic LBP [32]. Such controversy necessitates more investigation of acupuncture and its use for LBP to be conducted. To date, there has been no report on lumbar surgery rates of LBP patients who received acupuncture treatment vs. those who did not.

Health care in South Korea is provided by the National Health Insurance Service (NHIS). The enrollment rate has increased markedly since the 1980s, with about 97.2% of the entire Korean population receiving national health insurance benefits in 2013 [33]. In 2002, the NHIS created the NHIS Sample Cohort Database, which includes data of 1,025,340 subjects. The database was constructed with stratified sampling with regard to age, sex, and income, and thus adequately represents the whole Korean population [34]. The universal insurance coverage and stratified sampling help to avoid selection and participation bias. Furthermore, the longitudinal nature and large size of the database enable studies that require longer follow-up periods than typical trials. The database contains all records of acupuncture treatments and lumbar surgeries—items covered in the Korean health care system—without any recall bias which is often observed in self-reports from patients.

LBP may indicate a disorder in itself or a symptom manifesting as a result of other disorder(s). In this study, patients who were diagnosed of any disorder, with chief complaint of which includes but is not limited to LBP, were regarded as LBP patients. Through analysis of the NHIS Sample Cohort Database, the authors aimed to investigate whether acupuncture treatment is associated with any change in lumbar surgery rate for LBP patients in Korea.

## Methods

### Data source

The NHIS Sample Cohort Database for 2002–2013 was analyzed in the present study. All medical procedures performed in South Korea fall under either covered or non-covered items. In the case of covered items, incurred medical costs are co-payed by the NHIS and the patient. For reimbursement of the co-payment, each medical institution submits insurance claims reports to the NHIS containing detailed information about the services provided. Through compilation of such claims reports, the NHIS Sample Cohort Database was created for research purposes. Subjects who are lost due to death, immigration, or disqualification are replaced with newborns. The database contains information on claims reports, hospital billing, disease and injury reports, and prescription reports. Where applicable, each item of information is classified into medicine, Korean medicine, dentistry, or pharmacy. Prior to public release, patients’ personal information has been de-identified by the NHIS.

### Study design

**Fig 1:**
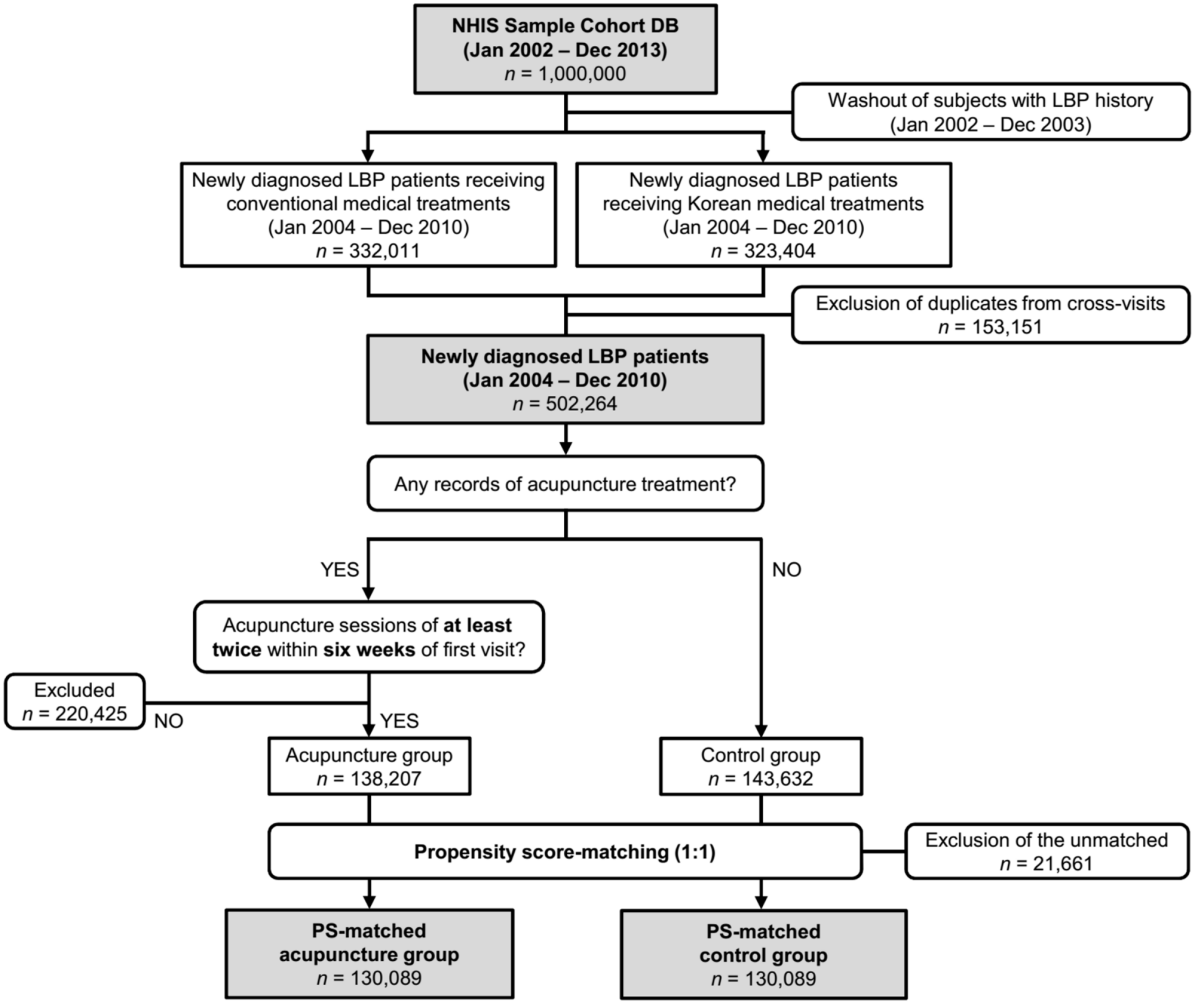
Schematic of the study design. NHIS Sample Cohort DB, National Health Insurance Service Sample Cohort Database; LBP, low back pain; PS, propensity score.

The period from January 1, 2002 to December 31, 2003 was set as the wash-out period to discard LBP subjects with possible medical history that cannot be identified within the dataset. Only the records of patients newly diagnosed with LBP were then identified from January 1, 2004 to December 31, 2010. The disorders of interest were those with LBP as the major symptom. Based on a previous study [35] and a discussion among the investigators, the following diseases and injuries were selected: M43* (deforming dorsopathies), M47* (spondylosis), M48* (spondylopathies), M51* (intervertebral disc disorders), M54* (dorsalgia), M99* (biomechanical lesions), and S33* (dislocation, sprain, and strain of joints and ligaments of the lumbar spine and pelvis). The selected disease codes were comparable with those selected in similar literatures [35–37] (S1 Table). Before the Korean Classification of Disease was unified in 2010 to be adopted by all conventional medicine and Korean medicine practitioners, Korean medicine doctors had used an independent disease classification system up to 2009. Therefore, J10* (LBP and lower extremity pain) and H354 (injury of soft tissue component, lumbar) were searched for for LBP treatments given by Korean medicine practitioners until December 31, 2009; M43* through S33* were searched alike thereafter. Patients with the above disorders as either primary or secondary diagnoses were defined as LBP subjects. Given that patients may cross-visit conventional medical and Korean medical facilities, duplicate cases were removed using their unique identification number, which was randomly generated only to be used within the dataset for identification purposes. The total number of LBP patients in Korea was thus calculated as the sum of those visiting either type of medical institution. The patients were then divided into an acupuncture group and a control group, for comparison of lumbar surgery rates (Fig 1).

### Group assignment

Patients who had no record of acupuncture treatment after the first LBP-related visit were assigned to the control group. The acupuncture intervention was defined as at least two acupuncture sessions within six weeks of the first LBP-related visit, as the authors contemplated that (1) a single session would not be sufficient to be analyzed as an acupuncture case, and (2) sporadic visits for treatment may imply that the subject is not a true case of LBP. Only those who met this definition were included in the acupuncture group; the rest who did not were discarded. Acupuncture treatment was identified using the codes 40011* (acupuncture, single body part) and 40012* (acupuncture, multiple body parts), which are mutually exclusive.

### Propensity score matching

Adjusting for disease severity in each study group was considered critical in the plan to assess the potential association between acupuncture therapy and the likelihood of lumbar surgery. However, due to the nature of the data included in the NHIS Sample Cohort Database, it was difficult to infer information about the severity of a patient’s disease from the claims report as no imaging data, such as magnetic resonance images, were available. Furthermore, data on specific modalities, e.g., medication and injections, could not be used for matching as they were prescribed after the first visit. In this study, we used propensity score matching to adjust for the two groups. Propensity scores indicate the patients’ propensity to choose a certain type of treatment, and may be utilized to construct similar control groups in observational studies [38]. In this study, the propensity score was calculated based on sex, age, income level, and Charlson Comorbidity Index (CCI); 1 to 1 matching was conducted taking into account the size of each group. Subjects not included in the 1 to 1 matching in each group were removed. The original data presented age as a categorical value of 5-year age intervals; for the current study, subjects were re-assigned to 10-year age intervals, as have been by Kim et al. [39] To achieve an even size across all income groups, income level was defined as ‘upper’ in the top three deciles, ‘lower’ in the bottom three deciles, and ‘middle’ in the rest. The CCI was computed according to the international classification of disease, tenth version (ICD-10) using the methodology devised by Quan et al. [40]. Existing comorbidity was identified if a corresponding diagnosis code was present in the patient’s medical record within the year prior to the date of first LBP-related visit.

### Outcome variables and additional analysis

Propensity score-matched subjects in the acupuncture group and control group were included in the final analysis, in which incidence of lumbar surgery was the outcome of interest. Based on a previous study [35] and authors’ discussion, lumbar surgery was defined as N0444, N0445, N0446, N0447, N0453, N0466, N0469, N1493, N1494, N1495, N1496, and N1499 (Arthrodesis For Spinal Deformity, Vertebral Corpectomy, Arthrodesis of Spine-Lumbar Spine, Diskectomy (Invasive)-Lumbar Spine, Diskectomy By Endoscopy, Injection Procedure For Chemonucleolysis, Aspiration Procedure of Nucleus Pulposus of Intervertebral Disk and Laminectomy, Lumbar Spine). These codes, specific to Korea, were also comparable to similar studies conducted in Korea [35, 39, 41] (S2 Table). Using the same observation period after the first visit date in both study groups may introduce survivorship bias in favor of the acupuncture group. Therefore, the analysis period was set as two years after the first visit in the control group, and two years after the first acupuncture session in the acupuncture group. Occurrence of lumbar surgery during the period above was investigated, and the surgery rates in both groups were compared.

To ascertain the robustness of the analysis, both groups were additionally compared with stratified analyses according to age, sex, and income level variables. Sensitivity analyses were further conducted by modulating the numbers (i.e., the treatment window and number of sessions) defining the intervention, which was originally determined as at least two sessions within six weeks following the initial LBP-related visit.

### Statistical analysis

Demographic characteristics and surgery rates were compared between the study groups using chi-squared test or Student’s *t*-test. Logistic regression was used for propensity score matching. The Cox proportional hazards model was used to calculate hazard ratios (HRs) and 95% confidence intervals (CIs). The cumulative surgery rate in each group was computed using Kaplan-Meier analysis. The statistical analyses were conducted using SAS version 9.4 (SAS Institute Inc., Cary, NC, USA) and Stata 14 (StataCorp, College Station, TX, USA). A two-sided p-value <0.05 was regarded as statistically significant.

### Ethics statement

The current study was reviewed and qualified as an exemption by the Institutional Review Board of Jaseng Hospital of Korean Medicine, Seoul, Korea (JASENG 2017-11-001). As the study analyzed publicly available data, no consent was obtained by the authors; all personal information was de-identified by the NHIS prior to public release. The principles expressed in the Declaration of Helsinki have been adhered to in the analysis.

## Results

### Demographic characteristics and propensity score matching

Between 2004 and 2010, a total of 502,264 patients were newly diagnosed with LBP. Of these patients, those with no records of acupuncture were assigned to the control group and those who met the pre-defined acupuncture treatment criteria (at least two sessions within six weeks) were assigned to the acupuncture group. All remaining subjects, i.e., subjects who had record(s) of acupuncture treatments but failed to suffice the above criteria, were removed. Finally, 138,207 patients were included in the acupuncture group and 143,632 patients in the control group.

Before matching, the two study groups significantly differed in age, sex, income, and CCI. The acupuncture group was generally older than the control group and the proportions of women and high-income subjects were higher in the acupuncture group. The CCI was also higher in the acupuncture group than in the control group (Table 1).

**Table 1:** Demographic characteristics of unmatched cohorts.

Characteristics	Acupuncture (*n* = 138,207)		Control (*n* = 143,632)		*p* value*	SMD (%)
	*n*	(%)	*n*	(%)		
*Age*						
20–29	21,736	15.73	26,515	18.46	<0.0001	-7.26
30–39	32,513	23.52	34,073	23.72		-0.47
40–49	34,633	25.06	35,360	24.62		1.02
50–59	14,383	10.41	14,659	10.21		0.66
60–69	20,215	14.63	19,867	13.83		2.29
70–79	14,727	10.66	13,158	9.16		5.02
*Sex*						
Male	64,677	46.80	70,448	49.05	<0.0001	-4.51
Female	73,530	53.20	73,184	50.95		4.51
*Income*						
Lower	30,526	22.09	33,596	23.39	<0.0001	-3.10
Middle	55,142	39.90	56,544	39.37		1.08
Upper	52,539	38.01	53,492	37.24		1.59
*CCI*						
0	77,676	56.20	82,823	57.66	<0.0001	-2.95
1	38,170	27.62	40,199	27.99		-0.83
2	15,081	10.91	14,482	10.08		2.71
3	5,076	3.67	4,405	3.07		3.33
4	1,593	1.15	1,258	0.88		2.69
5	459	0.33	346	0.24		1.69
6	115	0.08	85	0.06		0.76
7	29	0.02	26	0.02		0.00
8	7	0.01	8	0.01		0.00
9	1	0.00	0	0.00		NA

*p value from chi-square test

SMD, standardized mean difference; CCI, Charlson Comorbidity Index

The demographic characteristics of the patients in the study groups were then re-investigated after matching for age, sex, income, and CCI. After 1 to 1 matching, 130,089 subjects remained in each group. The post-matching groups showed no significant differences with regard to age, sex, income, or CCI (Table 2).

**Table 2:** Demographic characteristics of propensity score-matched cohorts.

Characteristics	Acupuncture (*n* = 130,089)		Control (*n* = 130,089)		*p* value*	SMD (%)
	*n*	(%)	*n*	(%)		
*Age*						
20–29	21,730	16.70	21,730	16.70	1.0000	0.00
30–39	31,264	24.03	31,264	24.03		0.00
40–49	33,958	26.10	33,958	26.10		0.00
50–59	13,600	10.45	13,599	10.45		0.00
60–69	17,811	13.69	17,811	13.69		0.00
70–79	11,726	9.01	11,727	9.01		0.00
*Sex*						
Male	63,265	48.63	63,265	48.63	1.0000	0.00
Female	66,824	51.37	66,824	51.37		0.00
*Income*						
Lower	29,252	22.49	29,251	22.49	1.0000	0.00
Middle	51,607	39.67	51,608	39.67		0.00
Upper	49,230	37.84	49,230	37.84		0.00
*CCI*						
0	75,377	57.94	75,378	57.94	1.0000	0.00
1	35,966	27.65	35,966	27.65		0.00
2	13,131	10.09	13,131	10.09		0.00
3	4,067	3.13	4,067	3.13		0.00
4	1,166	0.90	1,166	0.90		0.00
5	297	0.23	297	0.23		0.00
6	65	0.05	64	0.05		0.00
7	17	0.01	17	0.01		0.00
8	3	0.00	3	0.00		0.00

*p value from chi-square test

SMD, standardized mean difference; CCI, Charlson Comorbidity Index

### Overall and stratified analyses

**Fig 2:**
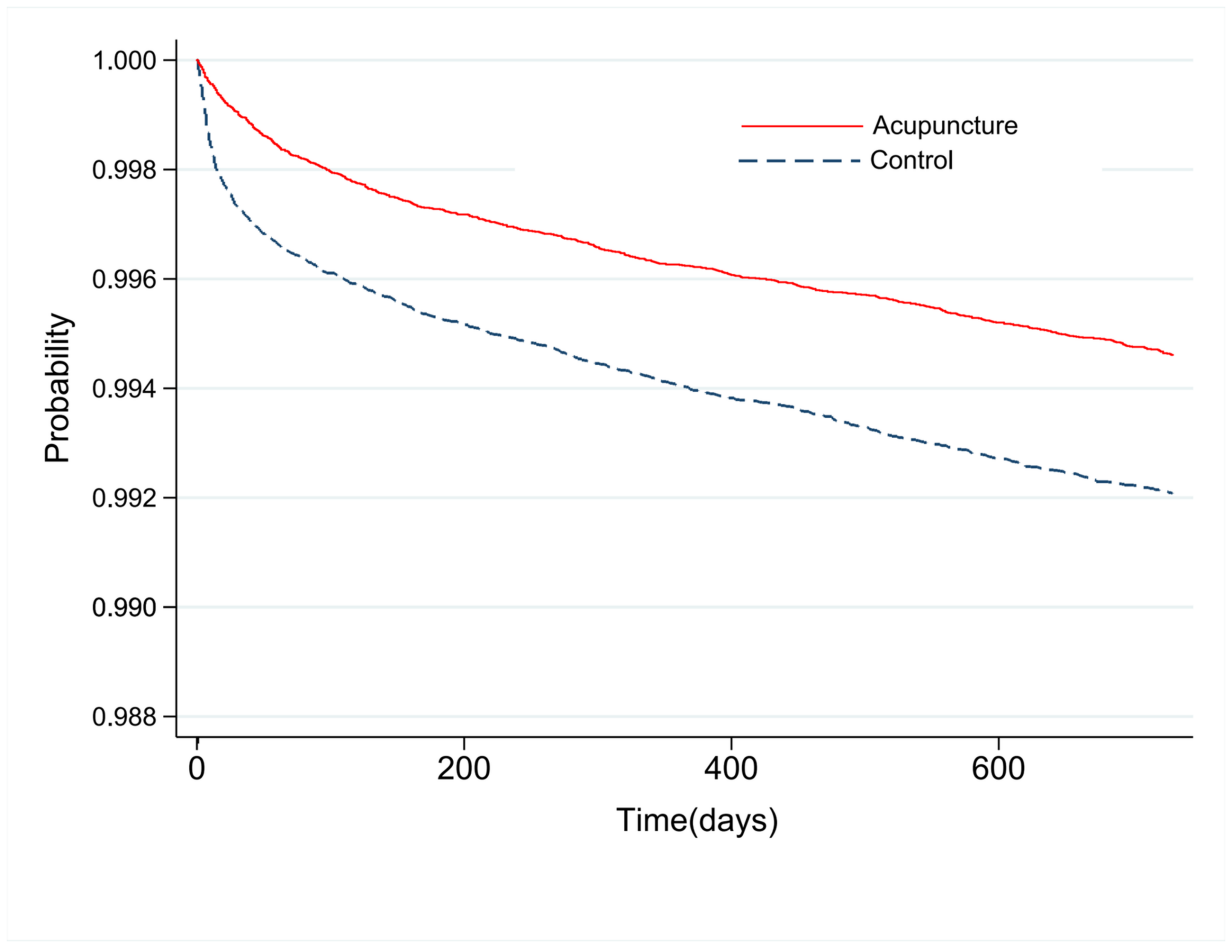
Survival analysis on lumbar surgery rate in acupuncture and control groups.

Lumbar surgery rates of the matched acupuncture group (followed-up to two years after the first acupuncture session) and the control group (followed-up to two years after the first visit) were compared. Seven hundred and one patients in the acupuncture group and 1,104 patients in the control group underwent lumbar surgery within the follow-up period, representing rates of 0.54% and 0.85%, respectively. The difference in likelihood of lumbar surgery between the acupuncture group and the control group was statistically significant (Control, ref; acupuncture group, HR 0.633, 95% CI 0.576–0.696). The result was also consistent prior to matching (HR 0.636, 95% CI 0.580–0.697). The lumbar surgery rates of the matched acupuncture and control groups are shown with Kaplan-Meier survival curves (Fig 2).

Next, stratified analyses were conducted to examine whether this difference was consistent across different age, sex, and income strata. While the HR for lumbar surgery in patients in their 20s was not statistically different between groups (HR 1.031, 95% CI 0.804–1.323), the acupuncture group had a significantly lower HR for lumbar surgery compared to the control group in all other age strata, i.e., 30s and older. In the sex-stratified analysis, the HR for lumbar surgery was lower in the acupuncture group for both men and women compared to the control group of the corresponding sex, respectively. Reduction of HR in the acupuncture group was greater in women than in men. Finally, in the income-stratified analysis, the HR was consistently lower in the acupuncture group than in the control groups for all three income strata. The HR in the acupuncture groups was further reduced in the upper income group than the middle or the lower groups (Table 3).

**Table 3:** Overall and stratified analyses on lumbar surgery rate in acupuncture and control groups.

	Acupuncture			Control			HR	95% CI	*p* value*
	*n*	cases	(%)	*n*	cases	(%)			
**Overall analysis**									
Unmatched	138,207	732	0.53	143,632	1,202	0.84	0.636	(0.580, 0.697)	<0.0001
Matched	130,089	701	0.54	130,089	1,104	0.85	0.633	(0.576, 0.696)	<0.0001
**Stratified analysis**									
*Age*									
20–29	21,730	126	0.58	21,730	122	0.56	1.031	(0.804, 1.323)	0.8077
30–39	31,264	138	0.44	31,264	216	0.69	0.637	(0.515, 0.789)	<0.0001
40–49	33,958	186	0.55	33,958	283	0.83	0.656	(0.545, 0.789)	<0.0001
50–59	13,600	76	0.56	13,599	122	0.90	0.621	(0.467, 0.827)	0.0011
60–69	17,811	101	0.57	17,811	213	1.20	0.473	(0.373, 0.599)	<0.0001
70–79	11,726	74	0.63	11,727	148	1.26	0.498	(0.377, 0.659)	<0.0001
*Sex*									
Male	63,265	449	0.71	63,265	670	1.06	0.668	(0.593, 0.753)	<0.0001
Female	66,824	252	0.38	66,824	434	0.65	0.579	(0.496, 0.677)	<0.0001
*Income*									
Lower	29,252	163	0.56	29,251	214	0.73	0.760	(0.620, 0.932)	0.0084
Middle	51,607	276	0.53	51,608	438	0.85	0.629	(0.541, 0.731)	<0.0001
Upper	49,230	262	0.53	49,230	452	0.92	0.578	(0.496, 0.673)	<0.0001

*p value from Cox-regression analysis adjusted by age, sex, income and CCI

HR, hazard ratio; CI, confidence interval; CCI, Charlson Comorbidity Index

### Sensitivity analysis

The overall and stratified analyses showed that acupuncture treatment was associated with a significantly lowered lumbar surgery rate; and that the result was consistent across all strata, except for subjects in their 20s. To test the robustness of the findings, we performed sensitivity analyses by modulating the definition of acupuncture treatment (at least two sessions within six weeks after the first visit for LBP). First, the required number of sessions was altered to be at least two, three, four, or five; the treatment window was fixed as six weeks following the initial visit. The results consistently support that HR for lumbar surgery in the acupuncture group is lower than that in the control group, with no particular trend with regard to the minimum number of sessions. Next, the treatment window was modulated as five, four, three, two, or one week(s) after the first visit; the minimum number of acupuncture sessions was fixed as two. While the results, again, consistently supported decreased HR for lumbar surgery in the acupuncture group, the HR showed a further decreasing trend as the treatment window was shortened (Table 4).

**Table 4:** Sensitivity analyses on lumbar surgery, according to the number of acupuncture sessions and the treatment period, in acupuncture and control groups.

	Acupuncture			Matched Control			HR	95% CI	*p* value*
	*n*	cases	(%)	*n*	cases	(%)			
**Number of sessions for acupuncture group**									
2 and more	130,089	701	0.54	130,089	1,104	0.85	0.633	(0.576, 0.696)	<0.0001
3 and more	109,149	600	0.55	109,149	926	0.85	0.646	(0.583, 0.716)	<0.0001
4 and more	93,830	524	0.56	93,830	786	0.84	0.665	(0.595, 0.743)	<0.0001
5 and more	81,629	454	0.56	81,629	692	0.85	0.654	(0.581, 0.737)	<0.0001
**Treatment period** (weeks) **for acupuncture group**									
1	126,802	595	0.47	126,802	1,074	0.85	0.552	(0.500, 0.611)	<0.0001
2	128,139	624	0.49	128,139	1,084	0.85	0.574	(0.520, 0.633)	<0.0001
3	128,891	656	0.51	128,891	1,095	0.85	0.598	(0.543, 0.659)	<0.0001
4	129,412	675	0.52	129,412	1,093	0.84	0.616	(0.559, 0.678)	<0.0001
5	129,772	693	0.53	129,772	1,101	0.85	0.628	(0.571, 0.690)	<0.0001

*p value from Cox-regression analysis adjusted by age, sex, income and CCI

HR, hazard ratio; CI, confidence interval; CCI, Charlson Comorbidity Index

## Discussion

The current study investigated whether administration of acupuncture treatment is associated with a change in lumbar surgery rate for LBP patients. Of all modalities used to treat and manage LBP, lumbar surgery tends to be accompanied by more complications (e.g., infection and failed back surgery syndrome), poses a greater financial burden than non-invasive procedures, and does not necessarily ensure successful outcomes [42]. A retrospective observational study of 200,000 patients in the U.S. reported that the mean direct cost for LBP patients was $7,211, whereas the mean direct cost of the subset who underwent surgery was $33,931 [43]. Non-invasive management of LBP, where appropriate, is therefore of heightened importance both for individuals and the overall health care system.

The present findings indicate that acupuncture treatment is associated with lower HR for lumbar surgery in LBP patients, and that the association was consistently observed across sexes, all income levels, and all age groups except for patients in their 20s. One interesting finding was that the acupuncture-associated decrease in HR was more evident in older age groups as it more than halved in patients aged 60 years or older. The surgery rate of patients in their 20s was about two-thirds of the overall rate. The acupuncture-associated decrease in HR may not have been observed in specific subsets as there was not much margin of improvement to begin with, whereas the opposite may be true for older populations. Contrasting etiology for different age groups (i.e., acute or traumatic for the young vs. chronic or degenerative for the aged) may also explain the phenomenon. In addition, difference in medical service preferences (i.e., conventional vs. Korean medicine) among various age groups, especially as observed in those in their 20s, may affect the choice of whether to receive surgery or not. It should be noted, however, that the number of patients aged in their 20s was similar to other age subsets in the acupuncture group. Although we could not infer any sex-related trend, the association between acupuncture and lumbar surgery seemed to be greater in women. Similarly, the association was greater in the upper income groups. In addition, high-income patients were observed to receive acupuncture therapy more often, prior to matching. As mentioned before, different preferences among various strata (i.e., men vs. women, high income group vs. others) may affect the patients’ choice to undergo surgery or not, or to receive acupuncture treatment or not. This may explain the observation that the percentages of women and high-income patients were higher in the before-matching acupuncture group, which phenomena were similarly observed in the U.S. and Canada as well [44, 45].

Sensitivity analyses were conducted by modifying the definition of acupuncture treatment to investigate the robustness of the results. When the intervention was defined as at least three, four, or five sessions with the treatment window fixed as six weeks, the decrease in the HR remained largely consistent across the number of sessions and revealed no particular trends. On the other hand, when the treatment window was shortened to five, four, three, two, or one week(s) with the number of sessions fixed as at least two, HR for lumbar surgery in the intervention group showed a further decreasing trend. To clarify, the shortened treatment window does not necessarily dictate that the treatment was terminated within the specific treatment window; rather, those who failed to suffice the minimum number of sessions, i.e., two, within the given treatment window were excluded from the analysis. Any treatment that occurred outside the treatment window did not affect the inclusion/exclusion status of the subject; only the sessions that occurred within the treatment window, i.e., six to one week(s), were of interest and determined whether the subject belonged to the intervention group or not. Hence, the modified definition of the acupuncture intervention with shortened treatment window may be interpreted as more intensive acupuncture care given within a shorter period of time. The latter finding, therefore, implies that the likelihood of lumbar surgery may be further lowered if active acupuncture treatment is provided more intensively following the onset of LBP.

The current results show that 0.69% of LBP subjects (1,805 cases out of 130,089 subjects) underwent surgery during the observed period whereas a similarly conducted study in U.S. soldiers reported a surgery rate of 1.94% (7,446 cases out of 383,586 subjects) [46]. As widely known, spinal surgery rates vary greatly across countries and regions, although the prevalence of spinal disorders is similar worldwide [47, 48], which may be explained by differences in medical service accessibility, economic development, clinician education, etc. It should be also noted, however, that direct comparison of the rates observed in this cohort study may not be appropriate as definition of disorders, observation period, and population characteristics may vary across studies. On the other hand, the current results indicate that the annual incidence of lumbar spinal surgery in Korea approximated to 25.8 cases per 100,000 (annualized total cases/total cohort subjects). The number is comparable to the 23.1 cases per 100,000 observed in Australia [49], but much less than the 164 cases per 100,000 (fusion only) observed in the U.S. where the spinal surgery rate is increasing alarmingly [50].

Two hypotheses may explain the present findings. The first is that acupuncture manages LBP effectively, thereby decreasing the need for lumbar surgery. The American College of Physicians recommend that non-drug treatments, including acupuncture, should precede drug therapy for acute and chronic LBP and that non-drug therapies with proven efficacy, such as acupuncture, should be performed in patients who do not show improvement using self-care measures [28]. While a number of literature reviews have concluded that acupuncture alleviates pain and improves function in acute and chronic LBP and that it can be used alone or in combination with other treatments [51–54], critics recommend against acupuncture claiming that the evidence is insufficient and the mechanism of action is unclear [55]. Future studies are nonetheless called for to draw stronger conclusions, as the subject still remains controversial due to the divide between advocates’ and critics’ points of view [29–31, 55].

The second hypothesis is that a multidisciplinary approach between conventional and Korean medicine may lower lumbar surgery incidence. An additional analysis was conducted to identify all LBP-related medical visits of subjects in both groups. The number of visits to each type of medical care, types of specialist visited, and frequency of treatments given were investigated. The results show that 52,508 subjects out of 130,089 patients in the acupuncture group (40.36%) also received conventional medical care for a similar number of visits compared to the control: an average 12.65 vs. 10.51, respectively. The types of specialists visited and types of care given were also comparable between the two groups (S3 Table). Therefore, it may be stated that the acupuncture group subjects were more likely to be provided with multidisciplinary care including usual conventional treatment, whereas the control subjects were only provided with conventional medical care. As reported by a cohort study in the U.S., multidisciplinary decision-making led to decreased incidence of lumbar spinal surgery [56]. Referral for surgery may be found unnecessary in second opinions given by different healthcare professionals [57, 58] and through a multidisciplinary approach, patients may be offered more diverse conservative treatments which should be sufficiently explored before surgery [6, 56]. The above hypotheses may explain the findings where acupuncture treatment for LBP was associated with less lumbar surgeries performed.

One of the strengths of this study is the use of a nationwide database to ensure national representativeness. The NHIS Sample Cohort Database was constructed to represent South Korea as a nation, as opposed to merely representing a particular region, sex, or age, and its validity has been established [38]. Further, more than 260,000 individuals were analyzed, contributing to the high reliability of the findings. Actual claims reports determined whether or not acupuncture and/or surgery were given. Thus, we did not have to rely on patient recall or personal accounts, thereby avoiding introduction of several sources of bias. We were able to search all cases of acupuncture treatment and surgeries without loss to follow-up.

A limitation of the study was that disease severity could not be matched between the study groups. In decision-making of surgical treatments, patient factors such as neurological symptoms, functional disability and objective defects of anatomical structures should be considered [6, 59]; the present database is comprised of claims data and thus does not contain such clinical factors, e.g., radiological evaluation findings, functional limitations, pain, etc. The possibility that subjects in the control group may have had higher pain and functional limitations, or *vice versa*, cannot be ruled out. Such limitations should be taken into consideration when interpreting the current results. However, each group included about 130,000 individuals, so the authors speculate that disease severity would be similar between study groups per the law of large numbers, but clear evidence to support this assumption is lacking. To address this problem, we performed propensity score matching and compared overall LBP-related medical service usage between the two groups (S3 Table). Prospective studies in the future are needed to investigate whether acupuncture affects lumbar surgery rates in patient samples with LBP of similar severity. Likewise, any difference in the severity between subjects who underwent surgery and those who did not, for both groups, could not be identified. Even though the number of acupuncture sessions received by those who underwent surgery and those who did not were similar (S4 Table), this finding does not necessarily indicate that they were in the same condition to consider surgical treatments. Furthermore, this study only investigated patients with newly diagnosed LBP. In clinical practice, the natural course of LBP may differ depending on whether it is acute or chronic and according to the presence of radicular pain. However, we could not predict the type of LBP at the time of onset based solely on a database containing data from claims reports. Moreover, the present database has been constructed from the claims report of insured items only; non-insured medical treatments are inherently beyond the scope of this database. While the subjects of both acupuncture and control groups may have been provided various non-insured medical treatments, any discrepancies in non-insured treatments between groups or subjects could not be sufficiently addressed. This issue should be regarded as an innate limitation characteristic of this database. Finally, the acupuncture treatment in this study was defined from a legal and administrative perspective in that acupuncture is a needling procedure conducted by Korean medical doctors who are licensed by the Korean government. While the definition reflects real-world clinical settings (i.e., the technique was chosen based on the practitioner’s education, professional experience, and preference), it may differ from strictly controlled acupuncture types used in clinical trial environments.

## Conclusion

The present study investigated whether acupuncture treatment is associated with lumbar surgery rates in patients with LBP by analyzing the NHIS Sample Cohort Database. As the results show, acupuncture treatment was associated with a significant decrease in HR for lumbar surgery, and the findings were consistent across all sex, income, and age groups with an exception of subjects in their 20s. This decrease tended to become greater with advancing age and in groups with higher incomes. Sensitivity analyses with adjustment for the acupuncture treatment period and frequency confirmed the robustness of the study findings, and suggested that intensive implementation of acupuncture after the onset of LBP was associated with a greater reduction in the likelihood of lumbar surgery. The findings of this nationwide, retrospective cohort study indicate that acupuncture is associated with significantly lowered lumbar surgery rates in patients with LBP.

## Supporting information

S1 Table Comparison of disease codes adopted for low back pain studies. MD, medical doctor; KMD, Korean medical doctor; LBP, low back pain.
(DOCX)

S2 Table Comparison of surgery codes adopted for low back pain studies in Korea. (DOCX)

S3 Table Overall LBP-related medical service usage of acupuncture and control groups. * Out of 130,089 patients in the acupuncture group, 52,508 subjects (40.36%) cross-visited conventional medical clinics as well as Korean medical clinics. ** The most representative results are shown; results are from accumulative visits. LBP, low back pain; SD, standard deviation; MD, medical doctor; KMD, Korean medical doctor.
(DOCX)

S4 Table Mean number of acupuncture sessions for subjects in the acupuncture group. *p value from independent t-test SD, standard deviation.
(DOCX)

## References

[1] World Health Organization. Priority diseases and reasons for inclusion. 2014.

[2] Hoy D, Bain C, Williams G, March L, Brooks P, Blyth F, et al A systematic review of the global prevalence of low back pain. Arthritis & Rheumatism. 2012;64(6):2028–37. 22231424 10.1002/art.34347

[3] Vos T, Abajobir AA, Abate KH, Abbafati C, Abbas KM, Abd-Allah F, et al Global, regional, and national incidence, prevalence, and years lived with disability for 328 diseases and injuries for 195 countries, 1990–2016: a systematic analysis for the Global Burden of Disease Study 2016. The Lancet. 2017;390(10100):1211–59. 10.1016/S0140-6736(17)32154-2 PMC5605509 28919117

[4] Hay SI, Abajobir AA, Abate KH, Abbafati C, Abbas KM, Abd-Allah F, et al Global, regional, and national disability-adjusted life-years (DALYs) for 333 diseases and injuries and healthy life expectancy (HALE) for 195 countries and territories, 1990–2016: a systematic analysis for the Global Burden of Disease Study 2016. The Lancet. 2017;390(10100):1260–344. 10.1016/S0140-6736(17)32130-X PMC5605707 28919118

[5] Airaksinen O, Brox J, Cedraschi C, Hildebrandt J, Klaber-Moffett J, Kovacs F, et al Chapter 4 European guidelines for the management of chronic nonspecific low back pain. European spine journal. 2006;15:s192–s300. doi: 10.1007/s00586-006-1072-1 16550448 10.1007/s00586-006-1072-1 PMC3454542

[6] Chou R, Loeser JD, Owens DK, Rosenquist RW, Atlas SJ, Baisden J, et al Interventional therapies, surgery, and interdisciplinary rehabilitation for low back pain: an evidence-based clinical practice guideline from the American Pain Society. Spine. 2009;34(10):1066–77. doi: 10.1097/BRS.0b013e3181a1390d 19363457 10.1097/BRS.0b013e3181a1390d

[7] Chou R, Baisden J, Carragee EJ, Resnick DK, Shaffer WO, Loeser JD. Surgery for low back pain: a review of the evidence for an American Pain Society Clinical Practice Guideline. Spine. 2009;34(10):1094–109. doi: 10.1097/BRS.0b013e3181a105fc 19363455 10.1097/BRS.0b013e3181a105fc

[8] Koes BW, van Tulder M, Lin C-WC, Macedo LG, McAuley J, Maher C. An updated overview of clinical guidelines for the management of non-specific low back pain in primary care. European Spine Journal. 2010;19(12):2075–94. doi: 10.1007/s00586-010-1502-y 20602122 10.1007/s00586-010-1502-y PMC2997201

[9] Savigny P, Kuntze S, Watson P, Underwood M, Ritchie G, Cotterell M, et al Low back pain: early management of persistent non-specific low back pain. London: National Collaborating Centre for Primary Care and Royal College of General Practitioners 2009;14. 20704057

[10] Manchikanti L, Candido KD, Benyamin RM, Hirsch JA, McGirt MJ, Parker SL. Response. Journal of Neurosurgery: Spine. 2014;22(3):2–3. 10.3171/2014.6.SPINE14605 25525961

[11] Xia X-P, Chen H-L, Cheng H-B. Prevalence of adjacent segment degeneration after spine surgery: a systematic review and meta-analysis. Spine. 2013;38(7):597–608. doi: 10.1097/BRS.0b013e318273a2ea 22986837 10.1097/BRS.0b013e318273a2ea

[12] Helgeson MD, Bevevino AJ, Hilibrand AS. Update on the evidence for adjacent segment degeneration and disease. The spine journal. 2013;13(3):342–51. doi: 10.1016/j.spinee.2012.12.009 23420004 10.1016/j.spinee.2012.12.009

[13] Martin BI, Mirza SK, Comstock BA, Gray DT, Kreuter W, Deyo RA. Reoperation rates following lumbar spine surgery and the influence of spinal fusion procedures. Spine. 2007;32(3):382–7. doi: 10.1097/01.brs.0000254104.55716.46 17268274 10.1097/01.brs.0000254104.55716.46

[14] Shapiro CM. The failed back surgery syndrome: pitfalls surrounding evaluation and treatment. Physical medicine and rehabilitation clinics of North America. 2014;25(2):319–40. doi: 10.1016/j.pmr.2014.01.014 24787336 10.1016/j.pmr.2014.01.014

[15] Maher C, Underwood M, Buchbinder R. Non-specific low back pain. Lancet. 2016 doi: 10.1016/S0140-6736(16)30970-9 . 27745712 10.1016/S0140-6736(16)30970-9

[16] Deyo RA, Mirza SK. Trends and variations in the use of spine surgery. Clinical orthopaedics and related research. 2006;443:139–46. 16462438 10.1097/01.blo.0000198726.62514.75

[17] Pumberger M, Chiu Y, Ma Y, Girardi F, Mazumdar M, Memtsoudis S. National in-hospital morbidity and mortality trends after lumbar fusion surgery between 1998 and 2008. J Bone Joint Surg Br. 2012;94(3):359–64. doi: 10.1302/0301-620X.94B3.27825 22371544 10.1302/0301-620X.94B3.27825

[18] Aakster C. Concepts in alternative medicine Health and Wellbeing: Springer; 1993 p. 84–93.

[19] Posadzki P, Watson LK, Alotaibi A, Ernst E. Prevalence of use of complementary and alternative medicine (CAM) by patients/consumers in the UK: systematic review of surveys. Clinical Medicine. 2013;13(2):126–31. doi: 10.7861/clinmedicine.13-2-126 23681857 10.7861/clinmedicine.13-2-126 PMC4952625

[20] Fouladbakhsh JM, Stommel M. Using the behavioral model for complementary and alternative medicine: the CAM healthcare model. Journal of Complementary and Integrative Medicine. 2007;4(1).

[21] Langevin HM, Churchill DL, Wu J, Badger GJ, Yandow JA, Fox JR, et al Evidence of connective tissue involvement in acupuncture. The FASEB journal. 2002;16(8):872–4. doi: 10.1096/fj.01-0925fje 11967233 10.1096/fj.01-0925fje

[22] Zhao Z-Q. Neural mechanism underlying acupuncture analgesia. Progress in neurobiology. 2008;85(4):355–75. doi: 10.1016/j.pneurobio.2008.05.004 18582529 10.1016/j.pneurobio.2008.05.004

[23] Clement-Jones V, Tomlin S, Rees L, Mcloughlin L, Besser G, Wen H. Increased β-endorphin but not met-enkephalin levels in human cerebrospinal fluid after acupuncture for recurrent pain. The Lancet. 1980;316(8201):946–9. 10.1016/s0140-6736(80)92106-6 6107591

[24] Furlan AD, Van Tulder MW, Cherkin D, Tsukayama H, Lao L, Koes BW, et al Acupuncture and dry‐needling for low back pain. The Cochrane Library. 2005. 10.1002/14651858.CD001351.pub2 PMC12145953 15674876

[25] Haake M, Müller H-H, Schade-Brittinger C, Basler HD, Schäfer H, Maier C, et al German Acupuncture Trials (GERAC) for chronic low back pain: randomized, multicenter, blinded, parallel-group trial with 3 groups. Archives of internal medicine. 2007;167(17):1892–8. doi: 10.1001/archinte.167.17.1892 17893311 10.1001/archinte.167.17.1892

[26] Manheimer E, White A, Berman B, Forys K, Ernst E. Meta-analysis: acupuncture for low back pain. Annals of internal medicine. 2005;142(8):651–63. 15838072 10.7326/0003-4819-142-8-200504190-00014

[27] Moritz S, Liu MF, Rickhi B, Xu TJ, Paccagnan P, Quan H. Reduced health resource use after acupuncture for low-back pain. The Journal of Alternative and Complementary Medicine. 2011;17(11):1015–9. doi: 10.1089/acm.2010.0619 22070438 10.1089/acm.2010.0619

[28] Qaseem A, Wilt TJ, McLean RM, Forciea MA. Noninvasive Treatments for Acute, Subacute, and Chronic Low Back Pain: A Clinical Practice Guideline From the American College of Physicians Noninvasive Treatments for Acute, Subacute, and Chronic Low Back Pain. Annals of internal medicine. 2017;166(7):514–30. doi: 10.7326/M16-2367 28192789 10.7326/M16-2367

[29] Bernstein IA, Malik Q, Carville S, Ward S. Low back pain and sciatica: summary of NICE guidance. BMJ. 2017;356:i6748 doi: 10.1136/bmj.i6748 28062522 10.1136/bmj.i6748

[30] Trinh KV, Diep D, Dorsher P. A Critical Look into the 2016 NICE Guidelines: Acupuncture for Low-Back Pain and Sciatica. Medical Acupuncture. 2017;29(1):20–4.

[31] MacPherson H. NICE for some interventions, but not so NICE for others: Questionable guidance on acupuncture for osteoarthritis and low-back pain. The Journal of Alternative and Complementary Medicine. 2017;23(4):247–8. doi: 10.1089/acm.2017.0029 28304178 10.1089/acm.2017.0029

[32] Dagenais S, Tricco AC, Haldeman S. Synthesis of recommendations for the assessment and management of low back pain from recent clinical practice guidelines. The Spine Journal. 2010;10(6):514–29. doi: 10.1016/j.spinee.2010.03.032 20494814 10.1016/j.spinee.2010.03.032

[33] Song SO, Jung CH, Song YD, Park C-Y, Kwon H-S, Cha BS, et al Background and data configuration process of a nationwide population-based study using the Korean National Health Insurance System. Diabetes & metabolism journal. 2014;38(5):395–403. 25349827 10.4093/dmj.2014.38.5.395 PMC4209354

[34] Lee J, Lee JS, Park S-H, Shin SA, Kim K. Cohort Profile: The National Health Insurance Service–National Sample Cohort (NHIS-NSC), South Korea. International journal of epidemiology. 2016:dyv319. 10.1093/ije/dyv319 26822938

[35] Lee SM, Han SK, Kim JH, Jang BH, Jeong CL, Son HJ, et al. [Pain-related effectiveness of injection therapy for chronic low back pain (in Korean)]. National Evidence-based Healthcare Collaborating Agency, 2010.

[36] Maribo T, Schiøttz-Christensen B, Jensen C, Jensen LD. Risks of permanent disability in low back pain patients associated with different job positions: a 5-year follow-up study. European Spine Journal. 2016;25(4):1211–8. doi: 10.1007/s00586-015-4118-4 26184720 10.1007/s00586-015-4118-4

[37] Lee J-H, Park H-J, Lee H, Song M-Y. Acupuncture for chronic low back pain: protocol for a multicenter, randomized, sham-controlled trial. BMC musculoskeletal disorders. 2010;11(1):118. 20540806 10.1186/1471-2474-11-118 PMC2896349

[38] Rosenbaum PR, Rubin DB. Constructing a control group using multivariate matched sampling methods that incorporate the propensity score. The American Statistician. 1985;39(1):33–8.

[39] Kim CH, Chung CK, Park CS, Choi B, Hahn S, Kim MJ, et al Reoperation rate after surgery for lumbar spinal stenosis without spondylolisthesis: a nationwide cohort study. The Spine Journal. 2013;13(10):1230–7. doi: 10.1016/j.spinee.2013.06.069 24017959 10.1016/j.spinee.2013.06.069

[40] Quan H, Sundararajan V, Halfon P, Fong A, Burnand B, Luthi J-C, et al Coding algorithms for defining comorbidities in ICD-9-CM and ICD-10 administrative data. Medical care. 2005:1130–9. 16224307 10.1097/01.mlr.0000182534.19832.83

[41] Kim CH, Chung CK, Park CS, Choi B, Kim MJ, Park BJ. Reoperation rate after surgery for lumbar herniated intervertebral disc disease: nationwide cohort study. Spine. 2013;38(7):581–90. doi: 10.1097/BRS.0b013e318274f9a7 23023591 10.1097/BRS.0b013e318274f9a7

[42] Sahin N, Karahan AY, Devrimsel G, Gezer IA. Comparison among pain, depression, and quality of life in cases with failed back surgery syndrome and non-specific chronic back pain. Journal of Physical Therapy Science. 2017;29(5):891–5. doi: 10.1589/jpts.29.891 28603366 10.1589/jpts.29.891 PMC5462693

[43] Ivanova JI, Birnbaum HG, Schiller M, Kantor E, Johnstone BM, Swindle RW. Real-world practice patterns, health-care utilization, and costs in patients with low back pain: the long road to guideline-concordant care. The Spine Journal. 2011;11(7):622–32. doi: 10.1016/j.spinee.2011.03.017 21601533 10.1016/j.spinee.2011.03.017

[44] Alwhaibi M, Sambamoorthi U. Sex differences in the use of complementary and alternative medicine among adults with multiple chronic conditions. Evidence-Based Complementary and Alternative Medicine. 2016;2016. 10.1155/2016/2067095 PMC4863098 27239207

[45] Metcalfe A, Williams J, McChesney J, Patten SB, Jetté N. Use of complementary and alternative medicine by those with a chronic disease and the general population-results of a national population based survey. BMC Complementary and Alternative Medicine. 2010;10(1):58. 20955609 10.1186/1472-6882-10-58 PMC2967501

[46] Kardouni JR, Shing TL, Rhon DI. Risk factors for low back pain and spine surgery: A retrospective cohort study in soldiers. American journal of preventive medicine. 2016;51(5):e129–e38. doi: 10.1016/j.amepre.2016.06.005 27476385 10.1016/j.amepre.2016.06.005

[47] Cherkin DC, Deyo RA, Loeser JD, Bush T, Waddell G. An international comparison of back surgery rates. Spine. 1994;19(11):1201–6. 8073310 10.1097/00007632-199405310-00001

[48] Rajaee SS, Bae HW, Kanim LE, Delamarter RB. Spinal fusion in the United States: analysis of trends from 1998 to 2008. Spine. 2012;37(1):67–76. doi: 10.1097/BRS.0b013e31820cccfb 21311399 10.1097/BRS.0b013e31820cccfb

[49] Harris IA, Dantanarayana N, Naylor JM. Spine surgery outcomes in a workers’ compensation cohort. ANZ journal of surgery. 2012;82(9):625–9. doi: 10.1111/j.1445-2197.2012.06152.x 22882708 10.1111/j.1445-2197.2012.06152.x

[50] Pannell WC, Savin DD, Scott TP, Wang JC, Daubs MD. Trends in the surgical treatment of lumbar spine disease in the United States. The Spine Journal. 2015;15(8):1719–27. doi: 10.1016/j.spinee.2013.10.014 24184652 10.1016/j.spinee.2013.10.014

[51] Zeng Y, Chung JW-y. Acupuncture for chronic nonspecific low back pain: an overview of systematic reviews. European Journal of Integrative Medicine. 2015;7(2):94–107.

[52] Yuan J, Purepong N, Kerr DP, Park J, Bradbury I, McDonough S. Effectiveness of acupuncture for low back pain: a systematic review. Spine. 2008;33(23):E887–E900. doi: 10.1097/BRS.0b013e318186b276 18978583 10.1097/BRS.0b013e318186b276

[53] Liu L, Skinner M, McDonough S, Mabire L, Baxter GD. Acupuncture for low back pain: an overview of systematic reviews. Evidence-Based Complementary and Alternative Medicine. 2015;2015. 10.1155/2015/328196 PMC4364128 25821485

[54] Dalamagka M. Systematic Review: Acupuncture in Chronic Pain, Low Back Pain and Migraine. J Pain Relief. 2015;4(195):2.

[55] Cummings M, Hróbjartsson A, Ernst E. Should doctors recommend acupuncture for pain? BMJ. 2018;360:k970 doi: 10.1136/bmj.k970 29514785 10.1136/bmj.k970

[56] Yanamadala V, Kim Y, Buchlak QD, Wright AK, Babington J, Friedman A, et al Multidisciplinary evaluation leads to the decreased utilization of lumbar spine fusion: An observational cohort pilot study. Spine. 2017;42(17):E1016–E23. doi: 10.1097/BRS.0000000000002065 28067696 10.1097/BRS.0000000000002065

[57] Epstein NE. Are recommended spine operations either unnecessary or too complex? Evidence from second opinions. Surgical neurology international. 2013;4(Suppl 5):S353 doi: 10.4103/2152-7806.120774 24340231 10.4103/2152-7806.120774 PMC3841934

[58] Epstein NE, Hood DC. “Unnecessary” spinal surgery: A prospective 1-year study of one surgeon’s experience. Surgical neurology international. 2011;2. 10.4103/2152-7806.82249 PMC3130462 21776403

[59] Carragee EJ. Persistent low back pain. New England Journal of Medicine. 2005;352(18):1891–8. doi: 10.1056/NEJMcp042054 15872204 10.1056/NEJMcp042054

