# Supplementary material for: Impact of acupuncture treatment on the lumbar surgery rate for low back pain in Korea: A nationwide matched retrospective cohort study
Source: PLoS One. 2018 Jun 12;13(6):e0199042. doi: 10.1371/journal.pone.0199042 (PMC5997340; doi:10.1371/journal.pone.0199042)
Supplement: S1 Table — MD, medical doctor; KMD, Korean medical doctor; LBP, low back pain. (DOCX) [file pone.0199042.s001.docx]

**S1 Table. Comparison of disease codes adopted for low back pain studies**

| **Current study** | **Lee et al., 2010 [31]** | **Maribo et al., 2016 [32]** | **Lee et al., 2010 [33]** |
| --- | --- | --- | --- |
| *Codes used by MDs, and KMDs after 2010* |  | | |
| M43*  deforming dorsopathies | M43* | M43.0, 1, 2, 5, 8, 9 |  |
| M47*  spondylosis | M47* | M47.0-2, 8, 9 |  |
| M48*  spondylopathies | M48* | M48.0 |  |
| M51*  intervertebral disc disorders | M51* | M51.0, 1, 2, 3, 9 | M51.3 |
|  |  | M53.3, 8, 9 |  |
| M54*  dorsalgia | M54* | M54.1, 3, 4, 5, 6, 8, 9 | M54.5, 8, 9 |
|  |  | M96.1 |  |
| M99*  biomechanical lesions | M99* | M99.1, 3-6 |  |
| S33*  dislocation, sprain, and strain of joints and ligaments of the lumbar spine and pelvis | S33* |  | S33.5, 6, 7 |
| *Codes used by KMDs before 2010* |  | | |
| J10* (LBP and lower extremity pain) |  |  |  |
| H354 (injury of soft tissue component, lumbar) |  |  |  |

MD, medical doctor; KMD, Korean medical doctor; LBP, low back pain
