# Supplementary material for: Impact of acupuncture treatment on the lumbar surgery rate for low back pain in Korea: A nationwide matched retrospective cohort study
Source: PLoS One. 2018 Jun 12;13(6):e0199042. doi: 10.1371/journal.pone.0199042 (PMC5997340; doi:10.1371/journal.pone.0199042)
Supplement: S2 Table — (DOCX) [file pone.0199042.s002.docx]

**S2 Table. Comparison of surgery codes adopted for low back pain studies in Korea**

| **Current study** | **Lee et al., 2010 [31]** | **Kim et al., 2013 [35]** | **Kim et al., 2013 [37]** |
| --- | --- | --- | --- |
| N0444-0447  Arthrodesis For Spinal Deformity | N0444-0447 |  |  |
| N0453  Vertebral Corpectomy | N0453 |  |  |
| N0466  Fusion, anterior | N0466 | N0466 | N0466 |
| N0469  Fusion, posterior | N0469 | N0469 | N0469 |
| N1493-1494  Discectomy | N1493 | N1493-1494 | N1493-1494 |
| N1495-1496  Nucleolysis | N1495-1496 | N1495-1496 | N1495-1496 |
| N1499  Laminectomy | N1499 | N1499 | N1499 |
