# Supplementary material for: Impact of acupuncture treatment on the lumbar surgery rate for low back pain in Korea: A nationwide matched retrospective cohort study
Source: PLoS One. 2018 Jun 12;13(6):e0199042. doi: 10.1371/journal.pone.0199042 (PMC5997340; doi:10.1371/journal.pone.0199042)
Supplement: S3 Table — * Out of 130,089 patients in the acupuncture group, 52,508 subjects (40.36%) cross-visited conventional medical clinics as well as Korean medical clinics. ** The most representative results are shown; results are from accumulative visits. LBP, low back pain; SD, standard deviation; MD, medical doctor; KMD, Korean medical doctor. (DOCX) [file pone.0199042.s003.docx]

**S3 Table. Overall LBP-related medical service usage of acupuncture and control groups**

|  |  | **Acupuncture (n=130,089)** | | **Control (n=130,089)** | |
| --- | --- | --- | --- | --- | --- |
| ***Number of visits*** | | mean ± SD | | mean ± SD | |
|  | MDs* | 12.65 ± 22.95 | | 10.51 ± 26.82 | |
|  | KMDs | 13.54 ± 29.57 | |  | |
| ***Types of Specialty***** | | n | % | n | % |
| MDs | Orthopedic surgery | 449,631 | 63.22 | 850,090 | 62.17 |
|  | Neurosurgery | 74,954 | 10.54 | 121,852 | 8.91 |
|  | Internal medicine | 45,260 | 6.36 | 110,917 | 8.11 |
| KMDs | Acupuncture and Moxibustion | 1,145,402 | 69.59 |  |  |
|  | Korean internal medicine | 473,776 | 28.79 |  |  |
| ***Types of care given***** | | n | % | n | % |
| MDs | Consultation | 700,773 | 40.05 | 1,342,693 | 40.62 |
|  | Physiotherapy | 455,662 | 26.04 | 847,095 | 25.63 |
|  | Injection | 257,890 | 14.74 | 509,258 | 15.41 |
| KMDs | Consultation | 1,631,776 | 47.41 |  |  |
|  | Procedures, acupuncture | 1,642,845 | 47.39 |  |  |

* Out of 130,089 patients in the acupuncture group, 52,508 subjects (40.36%) cross-visited conventional medical clinics as well as Korean medical clinics.

** The most representative results are shown; results are from accumulative visits.

LBP, low back pain; SD, standard deviation; MD, medical doctor; KMD, Korean medical doctor
