# Supplementary material for: Impact of acupuncture treatment on the lumbar surgery rate for low back pain in Korea: A nationwide matched retrospective cohort study
Source: PLoS One. 2018 Jun 12;13(6):e0199042. doi: 10.1371/journal.pone.0199042 (PMC5997340; doi:10.1371/journal.pone.0199042)
Supplement: S4 Table — *p value from independent t-test SD, standard deviation. (DOCX) [file pone.0199042.s004.docx]

**S4 Table.** Mean number of acupuncture sessions for subjects in the acupuncture group

|  | **Number of acupuncture sessions (mean ± SD)** | ***p* value*** |
| --- | --- | --- |
| Operated (n=701) | 13.93 ± 21.91 | 0.1378 |
| Not operated (n=129,388) | 12.64 ± 22.95 |  |

**p* value from independent t-test

SD, standard deviation
